# Role of Nucleolin in Endometrial Precancerous Hyperplasia and Carcinogenesis: Ex Vivo and In Silico Study

**DOI:** 10.3390/ijms23116228

**Published:** 2022-06-02

**Authors:** Vanya D. Barzilova, Josephine Drury, Bryony Rogers, Emily Thomas, Fareen Ahmed, Alice Bradfield, Hannan Al-Lamee, Dharani K. Hapangama

**Affiliations:** 1 Centre for Women’s Health Research, Department of Women’s and Children’s Health, Institute of Life Course and Medical Sciences, University of Liverpool, Liverpool L8 7SS, UK; psvbarzi@liverpool.ac.uk (V.D.B.); jadrury@liverpool.ac.uk (J.D.); b.c.v.rogers@liverpool.ac.uk (B.R.); e.l.thomas@liverpool.ac.uk (E.T.); f.ahmed8@liverpool.ac.uk (F.A.); hannan.al-lamee@liverpool.ac.uk (H.A.-L.); 2 Liverpool Women’s NHS Foundation Trust, Member of Liverpool Health Partners, Liverpool L8 7SS, UK; a.j.bradfield@liverpool.ac.uk; 3 Hewitt Centre for Reproductive Medicine, Liverpool Women’s NHS Foundation Trust, Liverpool L8 7SS, UK

**Keywords:** endometrial cancer, nucleolin, metastasis

## Abstract

Endometrial cancer (EC) is the most common gynaecological malignancy. Nucleolin (*NCL*) is involved in rDNA transcription, cell proliferation, and apoptosis, with high expression associated with worse overall survival (OS) in other adenocarcinomas. Our aims were to assess *NCL* gene and protein expression and explore the differential expression of *NCL*-associated genes (NAGs) in endometrial carcinogenesis. Endometrial samples were obtained from 157 women to include healthy, hyperplastic (EH), EC, and metastatic groups. RT-qPCR and immunohistochemistry were employed to assess *NCL* gene and protein levels. In silico analysis of NAGs in TCGA and GEO datasets was performed, with the prognostic value determined via Human Protein Atlas. *NCL* mRNA level of EC was lower than in healthy post-menopausal endometrium (*p* < 0.01). EH samples had lower NCL immuno-expression scores than healthy pre-menopausal (*p* < 0.001), benign post-menopausal (*p* < 0.01), and EC (*p* < 0.0001) samples. Metastatic lesions demonstrated higher NCL quick scores than primary tissue (*p* = 0.04). Higher NCL Immuno quick scores carried a worse OS in high-grade EC (*p* = 0.01). Interrogating Uterine Corpus Endometrial Carcinoma (TCGA-UCEC) and Uterine Carcinosarcoma (TCGA-UCS) cohorts revealed *NCL* to be the most highly upregulated gene in carcinosarcoma, with *S100A11*, *LMNB2*, *RERG*, *E2F1* and *CCNA2* representing key dysregulated NAGs in EC. Since *NCL* is implicated in transforming hyperplastic glands into cancer, with further involvement in metastasis, it is suggested to be a promising target for better-informed diagnosis, risk stratification, and management of EC.

## 1. Introduction

Endometrial cancer (EC) is the most common gynaecological malignancy, and the fourth most common cancer in women in the United Kingdom (UK) [1]. Incidence rates of EC are continuing to rise, with a reported increase of 55% since the early 1990s [1], attributed to increasing rates of obesity and an ageing population worldwide. Despite advances in current treatment strategies, EC mortality rates have increased by 25% in the last decade and are projected to rise a further 19% by 2035 [1]. This is of concern since there is a general improvement in overall survival for other gynaecological cancers, such as ovarian and cervical cancer [2,3]. Therefore, new therapeutic approaches are urgently needed to improve overall patient survival and curb the escalating burden of EC. New potential targets that play an important role in the aetiology, carcinogenesis, and tumour progression in EC are presently sought for the development of novel screening or treatment modalities for EC.

Immunotherapy is a new promising therapeutic approach that targets specific molecular markers. One such target could be nucleolin (*NCL*), which is a multifunctional protein found mainly in the nucleolus, with a role in rDNA transcription, cell proliferation, apoptosis, and angiogenesis [4,5,6]. Its overexpression has been noted in several cancer types, including colorectal, gastric, oesophageal, pancreatic, and hepatocellular cancers [7,8,9,10,11], with higher levels linked to a worse prognosis. The oncogenic properties of *NCL* can be explained by the increased mitotic activity of cancerous cells, which require a high level of protein synthesis to sustain mitosis. Dysregulated *NCL* increases rRNA and ribosomal synthesis, as well as increasing cell survival due to its anti-apoptotic properties [12], thereby contributing to malignant transformation, tumour migration, and distant metastasis. Despite the vast existing knowledge of *NCL*’s role in carcinogenesis relevant to many other cancers, its role in EC is yet to be fully established.

The human endometrium is a highly proliferative organ [13]. EC is associated with aberrant and dysregulated endometrial epithelial proliferation, coupled with errors in apoptotic and DNA repair pathways leading to an increased risk of tumour metastasis, a process known to be a key factor in cancer-associated mortality and poor survival in EC patients. Therefore, it is vital to explore the role of cancer-associated proteins, such as *NCL*, to establish their role in endometrial carcinogenesis.

The only study examining *NCL* in EC was published recently and analysed publicly available TCGA-UCEC RNA sequencing dataset of 494 endometrioid, serous, and mixed serous and endometrioid EC samples, suggesting that higher expression of *NCL* was an unfavourable prognostic factor [14]. They also examined the *NCL* protein levels in a limited cohort of 82 endometrioid ECs with immunohistochemistry and reported that low nuclear *NCL* and contrastingly high extra-nuclear *NCL* protein levels are associated with poor disease-free survival rates. This study did not include all subtypes of EC, particularly excluded non-endometrioid types of ECs that carry a worse prognosis than endometrioid EC subtype, and included a heterogeneous group of control, a non-malignant disease for comparison. Therefore, to fill the existing gaps in knowledge, our study aimed to examine *NCL* mRNA and protein expression in tissue samples from pre-menopausal and post-menopausal (PM) healthy endometrium, precancerous endometrial hyperplasia (EH), and all subtypes of EC, including endometrioid, serous, carcinosarcoma and clear cell subtypes, as well as metastatic lesions, thereby comprehensively examining differential levels of *NCL* in endometrial carcinogenesis. We hypothesised that the *NCL* gene and protein would be differentially expressed in EC.

Considering the diverse role of *NCL*, we generated a list of nucleolin-associated genes (NAGs) and examined their differential expression in TCGA EC datasets since they may allow the identification of important other targets and pathways relevant to prognosis and treatment in EC.

## 2. Results

### 2.1. Demographic Data

Women in the pre-menopausal group were younger than all other patient groups examined (*p* < 0.0001) (Table 1), and patients with EH were significantly younger than patients with EC (*p* = 0.0007), whilst having significantly higher BMI than women in the pre-menopausal (*p* = 0.0061), post-menopausal (*p* < 0.0001), and EC (*p* = 0.0008) patient groups.

### 2.2. NCL Gene Expression

**Figure 1:**
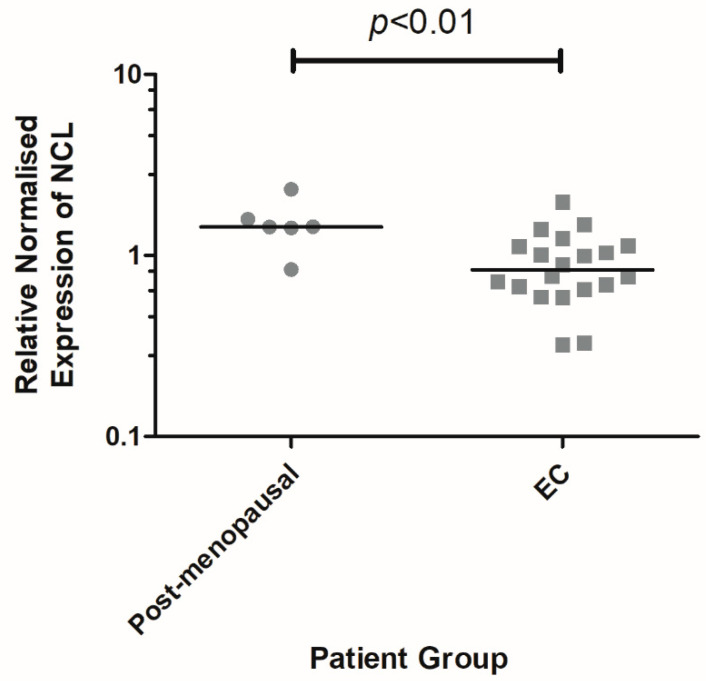
**Scatter plot of *NCL* mRNA expression using RT-qPCR in** post-menopausal (*n* = 6) versus endometrial cancer tissue (*n* = 20; G1 endometrioid *n* = 3, G2 endometrioid *n* = 3, G3 endometrioid *n* = 6, serous *n* = 2, carcinosarcoma *n* = 3, clear cell *n* = 3); line indicates the median.

Healthy PM endometrium expressed significantly higher levels of *NCL* mRNA compared with all EC samples (*p* < 0.01) (Figure 1).

### 2.3. Immunolocalisation of NCL

**Figure 2:**
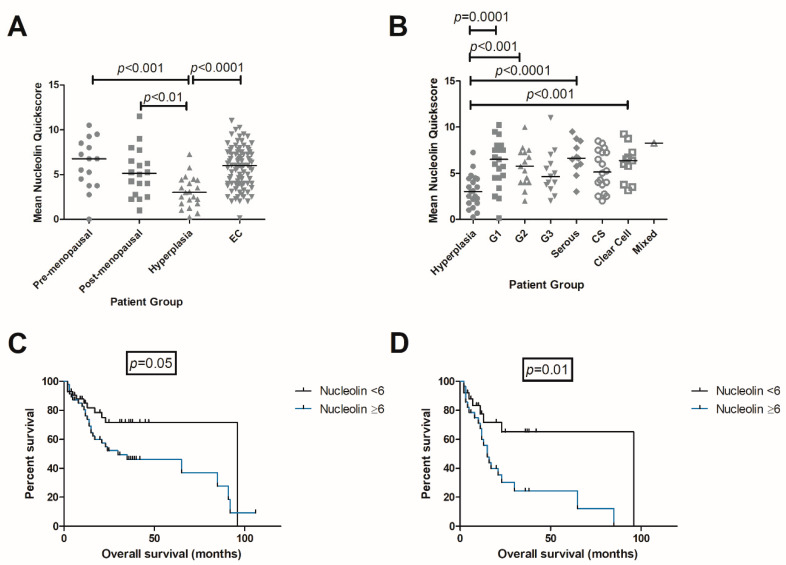
(**A**) Scatter plot showing *NCL* quickscore comparison across pre-menopausal (*n* = 15), post-menopausal (*n* = 18), EH (*n* = 21) and EC (*n* = 90) samples. Line indicates the median. (**B**) Scatter plot showing *NCL* quickscore comparison across EH (*n* = 21), G1 (*n* = 21), G2 (*n* = 15), G3 (*n* = 13), serous (*n* = 11), carcinosarcoma (*n* = 18), clear cell (*n* = 11), and mixed (*n* = 1) EC. Line indicates the median. (**C**) Kaplan–Meier survival curve showing effect of *NCL* expression on OS in EC. *p* = 0.05. (**D**) Kaplan–Meier survival curve showing effect of *NCL* expression on OS in HGEC. *p* = 0.01.

**Figure 3:**
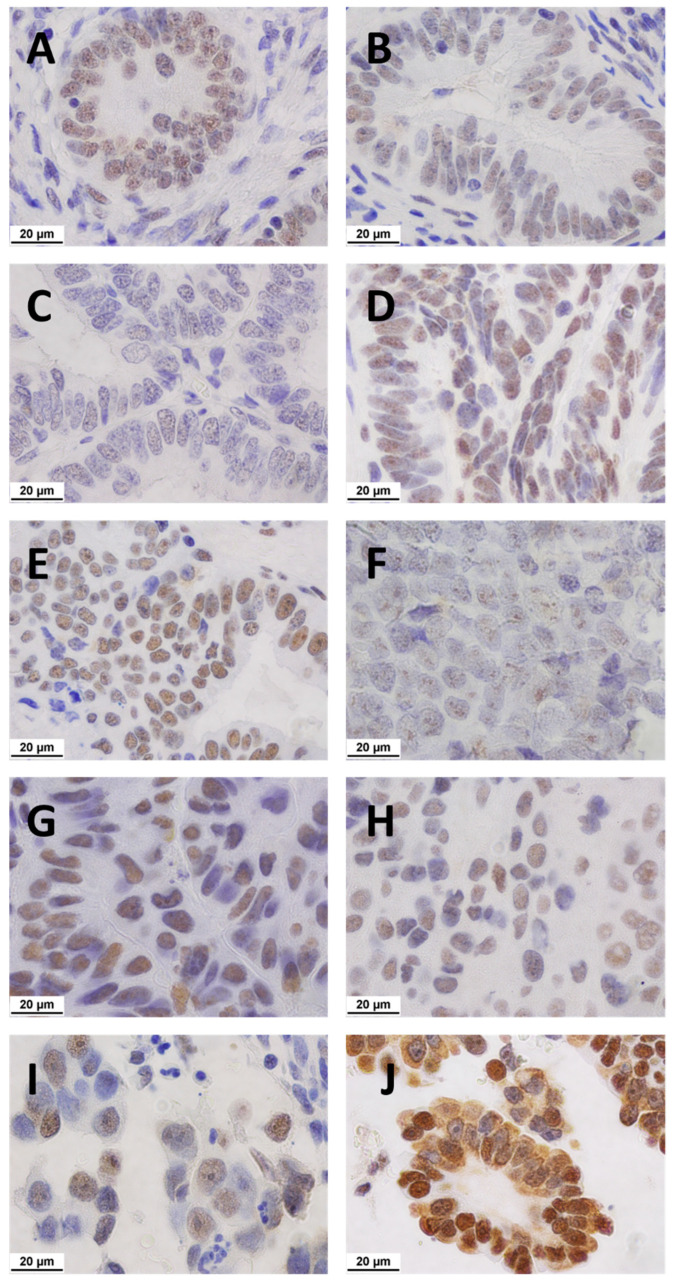
Representative microphotographs of immunolocalised nucleolar *NCL* expression in different tissues. Positive staining indicated by brown nucleoli. All images seen at ×1000 magnification. Scale bar = 20 μm, applicable to all panels. (**A**) Pre-menopausal (**B**) post-menopausal (**C**) hyperplasia (**D**) G1 endometrioid EC (**E**) G2 endometrioid EC (**F**) G3 endometrioid EC (**G**) serous EC (**H**) carcinosarcoma EC (**I**) clear cell EC (**J**) mixed. The median scores for each group were 6.8, 5.1, 2.8, 6.5, 5.8, 4.6, 6.6, 5.1, 6.4, and 8.3, respectively.

EH was associated with a significant reduction in *NCL* quick scores when compared with pre-menopausal endometrium (*p* < 0.001), post-menopausal endometrium (*p* < 0.01), and EC (*p* < 0.0001) (Figure 2A). Significantly higher *NCL* quick scores were observed in G1 endometrioid (*p* = 0.0001), G2 endometrioid (*p* < 0.001), serous (*p* < 0.0001), and clear cell EC (*p* < 0.001), when compared with EH (Figure 2B), with representative microphotographs presented in Figure 3.

Kaplan–Meier survival curves analyzing the overall survival (OS) were created to explore the prognostic value of *NCL* in EC. Several cut-off points were trialed and the score which provided the best overall separation was chosen, with a quick score of six. Although not statistically significant, there was an obvious trend with worse OS in women with EC expressing a high *NCL* quick score (≥6), than in women with low *NCL* quick score (<6) (*p* = 0.05) (Figure 2C), with median survival in those with a low *NCL* quick score (<6) at 96 months when compared with the 30 months median survival in those with high *NCL* quick score (≥6). Patients with high-grade EC (HGEC) tumours expressing high *NCL* quick score (≥6) had significantly poorer OS than patients with HGEC expressing lower *NCL* quick score (<6) (*p* = 0.01) (Figure 2D), and median survival in those with low *NCL* quick score (<6) at 96 months, when compared with 15 months median survival duration in those with high *NCL* quick score (>6).

#### 2.3.1. Matched Pairs Analysis of NCL Staining

**Figure 4:**
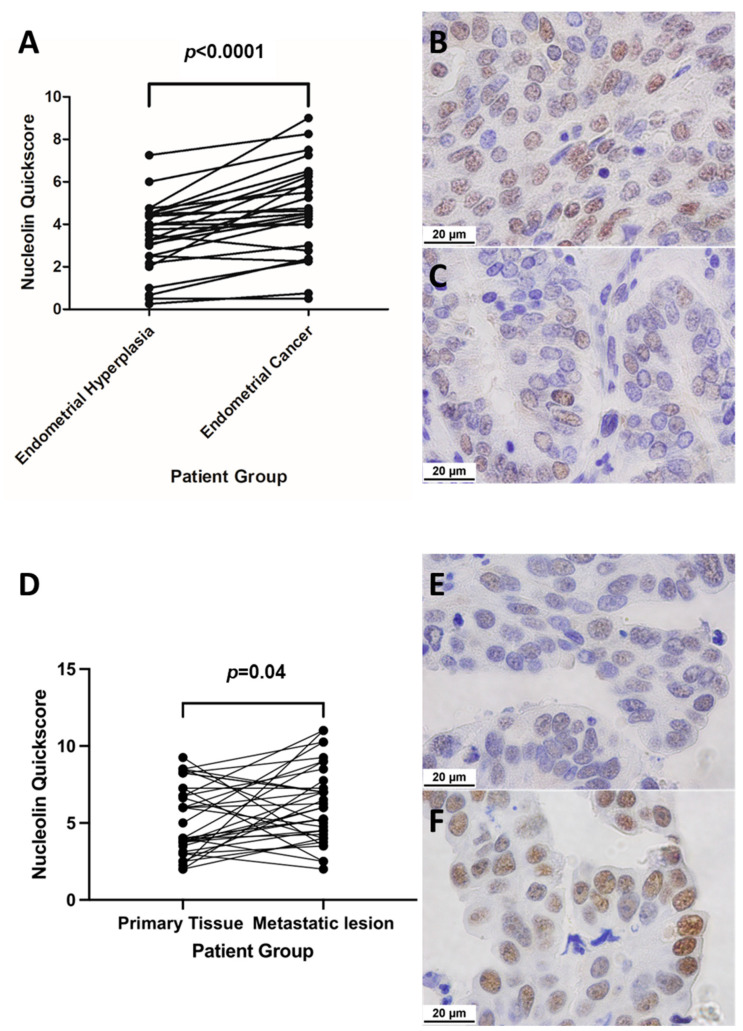
(**A**) Graph showing Wilcoxon matched pairs test in women diagnosed with both EH and EC (*n* = 26). Representative micrographs showing nucleolar *NCL* staining in (**B**) EC (median quick score = 4.6) and (**C**) matched EH (median quick score = 3.6). (**D**) Graph showing Wilcoxon matched pairs test in women diagnosed with metastatic EC, showing nucleolar *NCL* quick score in primary endometrial tissue (*n* = 27) and matched metastatic lesions (*n* = 35). Representative micrographs showing nucleolar *NCL* staining in (**E**) EC (median quick score = 4) and (**F**) matched metastatic lesion (median quick score = 6). Positive staining indicated by brown nucleoli. All images seen at ×1000 magnification. Scale bar = 20 μm, applicable to all panels.

Wilcoxon matched pairs test was carried out to compare *NCL* quick scores of women diagnosed to have both EH and EC on histology. Analysis of matched EH and EC samples from the same woman demonstrated a significant decrease in *NCL* immunolocalisation in EH when compared to EC (*p* < 0.0001) (Figure 4A) with representative micrographs of the immunolocalisation shown in Figure 4B(EC) and Figure 4C(hyperplasia). The second subgroup analysis compared the *NCL* quick scores of the metastatic EC, with the *NCL* quick scores of primary uterine cancer tissue. Figure 4D displays an overall higher nucleolar expression of *NCL* in the distant metastatic lesions when compared with primary cancer tissue from the uterine site of the same patient (*p* = 0.04). Figure 4E shows a G2 endometrioid EC, the most common subtype of EC, with the most common metastatic lesion for our subset—the omentum (Figure 4F).

#### 2.3.2. Observer Agreement

There was a high level of agreement between the three observers scoring the samples, as can be seen in the Bland–Altman plots between VB (observer A) and the three other observers, observers B (BR), C (FA), and D (ET) (Supplementary Figure S1).

### 2.4. In Silico Analysis

#### 2.4.1. NCL in TCGA Data

Demographics data of the TCGA Cohort is presented in Supplementary Table S1. Our analysis of TCGA data further reveals that *NCL* is upregulated in carcinosarcoma (Log2Fold Change = 6022.94), an HGEC subtype when compared to healthy adjacent endometrium (Supplementary Table S5).

#### 2.4.2. Identification of NAGs and DEGs

A total of 196 NAGs were identified via STRING and IPA (highest confidence = 0.900). Two additional NAGs of interest, namely *NAP1L1* and *SRFS2* (medium confidence = 0.400) were also included in the analysis due to their involvement in carcinogenesis and cancer metastasis [15,16,17]. The link between *NAP1L1* and *NCL*, as well as *SRFS2* and *NCL*, was based on co-expression and experimental data. A total of 198 NAGs were analysed for differential gene expression (Supplementary Table S2). As TCGA data were available for 197 of the 198 NAGs (excluding *TCR*), overall, 197 NAGs were included for differentially expressed genes (DEG) analysis.

A total of 52 DEGs were identified between EC (*n* = 120) and healthy adjacent control (*n* = 10). A total of 32 were upregulated in cancer and 20 downregulated (Supplementary Table S3). Supplementary Figure S2 depicts the volcano plots and heatmap of this analysis. The top five upregulated DEGs, ranked by Log2FC, were *S100A11*, *LMNB2*, *SCRIB*, *CCNB1,* and *SFRS2*. The top five downregulated DEGs were *SVIL*, *RERG*, *ITGAV*, *RASL12*, *GEM*, and *CDH5*.

**Figure 5:**
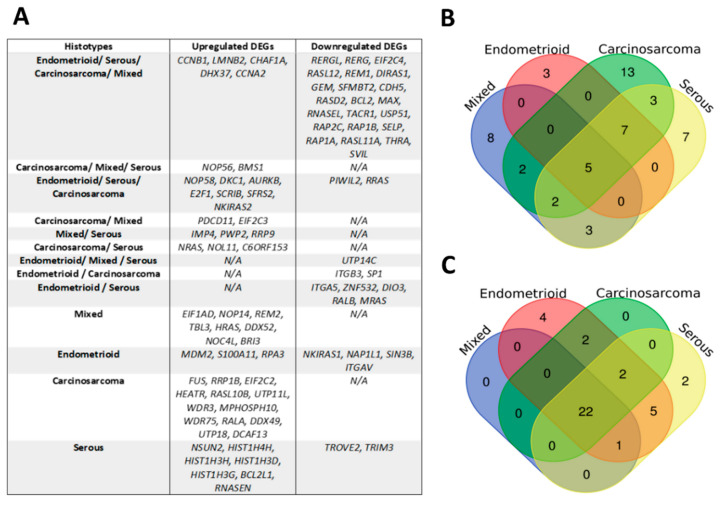
(**A**) Table of DEGs commonly upregulated and downregulated between EC subtypes. Venn diagrams displaying common (**B**) upregulated and (**C**) downregulated genes between each subtype.

Our analysis identified several NAGs (*n* = 27) common to all EC subtypes, as well as genes specific to each subtype, as seen in Figure 5, full tables available in Supplementary Tables S4–S7. Interestingly, *NCL* was the most highly upregulated DEG for the carcinosarcoma subtype (log_2_ Fold Change = 6022.939, Adjusted *p* value = 0.00609). Similarly, Supplementary Tables S8–S10 depict the common genes between EC grades and those specific to low-grade EC (LGEC) and HGEC.

As endometrioid tumours represent the most common histological subtype of EC, this study further examined DEGs amongst its different grades (Supplementary Figure S3 and Tables S11–S13).

#### 2.4.3. Identification of Prognostic DEGs

**Figure 6:**
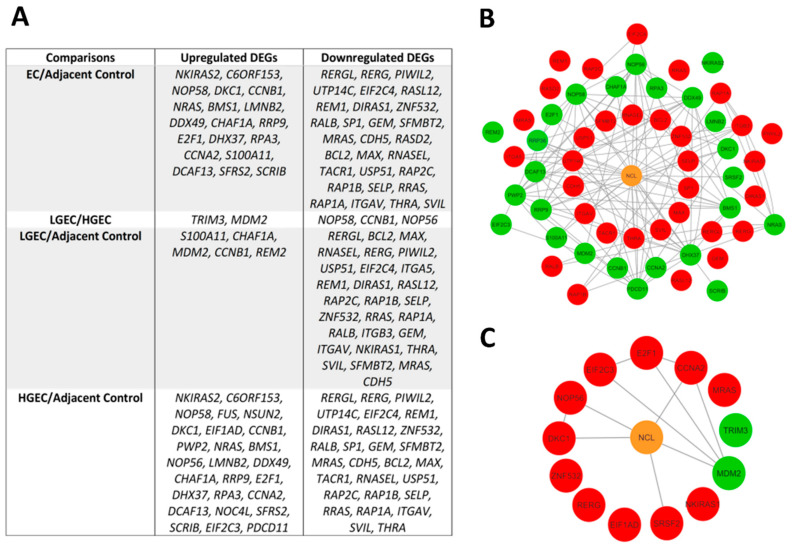
(**A**) Table of DEGs commonly upregulated and downregulated between those exposed and not exposed to hormonal, radiation, or neoadjuvant therapy. (**B**) Protein–protein interaction network displaying upregulated and downregulated DEGs in those exposed and not exposed to hormonal, radiation, or neoadjuvant therapy. Green and red nodes represent upregulated and downregulated DEGs, respectively. (**C**) Protein–protein interaction network of favourable (green) and unfavourable (red) genes in endometrial cancer.

The main sample set in this study included 130 samples not exposed to any radiation, neoadjuvant or hormonal therapy, whilst the larger TCGA cohort comprised 248 samples, including those exposed to the treatments. DEGs of the latter can be seen in Supplementary Tables S14–S16, with those common to both datasets in Figure 6. The prognostic value of 62 DEGs persisting despite hormonal, radiation, or neoadjuvant therapy was searched via The Human Protein Atlas. A total of 11 genes were found to be unfavourable in EC, namely *E2F1*, *CCNA2*, *DKC1*, *ZNF532*, *NOP56*, *SRSF2*, *RERG*, *MRAS*, *NKIRAS1*, *EIF2C3*, and *EIF1AD*, whilst 2 were favourable—*TRIM3* and *MDM2* (Figure 6C).

#### 2.4.4. External Validation of Differentially Expressed NAGs

The GSE17025 GEO dataset [18] was selected for DEG analysis due to its similarity to our local cohort with 63 Stage I LGEC (G1 and G2 Endometrioid), 25 Stage I HGEC (G3 Endometrioid and Serous), and 8 postmenopausal (atrophic and inactive endometrium) control samples. Supplementary Table S17 show the DEGs common to both TCGA and GEO datasets, thereby confirming this study’s findings.

#### 2.4.5. Biological Processes and Pathway Analyses

**Figure 7:**
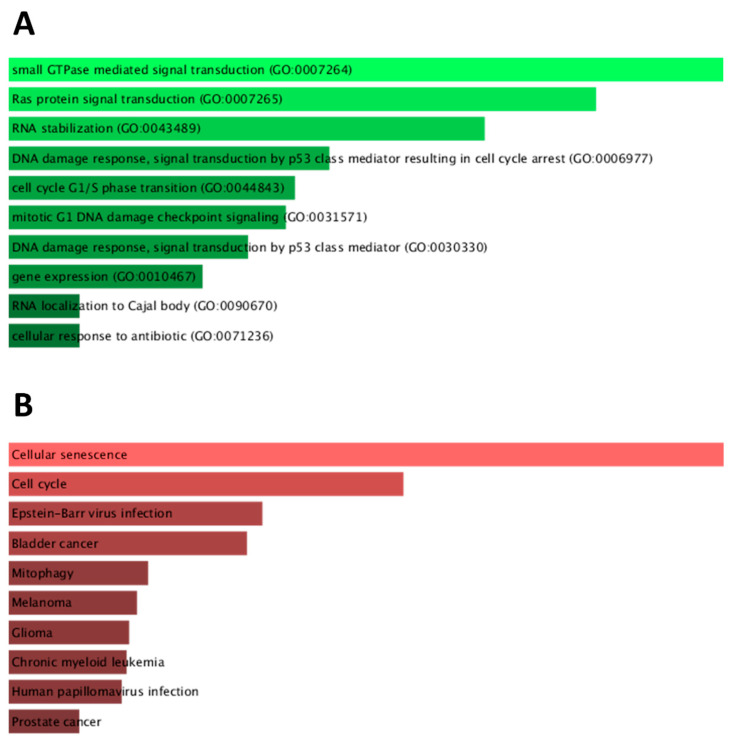
**Bar charts of** (**A**) biological processes and (**B**) KEGG pathways analyses in prognostic DEGs.

Analyses of biological processes and KEGG pathways were performed on the 13 prognostic DEGs shown in Figure 6C. A total of 180 significant GO biological processes and 50 KEGG pathways were identified (Supplementary Tables S18 and S19). Figure 7 depicts the ten highest ranking processes and pathways by *p*-value, as taken from Enrichr, with significant enrichment for cellular senescence and cell cycle through biological processes such as small GTPase mediated and Ras protein signal transduction, along with RNA stabilisation.

## 3. Discussion

Although there is a growing evidence base of molecular alterations in EC, many questions remain unanswered. The role of *NCL* in EC has not been fully elucidated. Nucleolin is a ubiquitous protein involved in cell growth and proliferation. Several studies have confirmed its relevance in cancer development [6]; however, there is limited data on nucleolin’s involvement in the malignant transformation of the endometrium [14]. We have previously immunolocalised *NCL* in the endometrium of healthy pre-menopausal women, with high expression observed in the proliferative phase of the menstrual cycle and reduced or absent *NCL* in the mid/late secretory phase, suggesting nucleolin expression may either be a feature of or play a facilitatory role in endometrial cell proliferation [13]. In this study, we sought to investigate the relationship between *NCL* expression and the human endometrium, including endometrial hyperplasia, primary cancerous tissue, and metastatic lesions.

This study is the first to examine the nucleolar expression of *NCL* in endometrial hyperplasia. EH is defined as an aberrant proliferation of endometrial glands with an increase in the gland-to-stroma ratio [19]. The most common histotype of EC, endometrioid EC, often originates from EH, with the risk of progression to cancer with atypical hyperplasia being as high as 30% [20]. Currently, there is no UK screening programme for women at risk of developing EC [21]. This highlights the need for identifying biomarkers to risk stratify and target early preventative treatment. In our study, we found EH to have an association with a reduction in *NCL* quick scores when compared to pre-menopausal endometrium (*p* < 0.001), post-menopausal endometrium (*p* < 0.01), and EC (*p* < 0.0001), with further analysis revealing lower *NCL* quick scores in EH were maintained when compared to G1 endometrioid (*p* = 0.0001), G2 endometrioid (*p* < 0.001), serous (*p* < 0.0001), and clear cell EC (*p* < 0.001). Analysis of matched EH and EC samples further supported our findings of a significant decrease in *NCL* immunolocalisation in EH when compared to EC (*p* < 0.0001). Our findings suggest that when hyperplastic glands undergo architectural and nuclear change typical of cancer, *NCL* expression is regained. This could suggest a possible involvement of *NCL* in the transformation of endometrial hyperplasia to endometrial cancer. This putative shift in the mechanistic role of nucleolin is important to explore and future laboratory studies utilising gene manipulation may allow examination of the effects of both over-expression or lack of *NCL* on endometrial carcinogenesis. Reintroducing *NCL*, after knock-down, may simulate the findings we report from this clinical observational study. Current statistics show that 34% of EC cases in the UK are preventable [1]. Further research into *NCL*’s role in the transformation of EH into EC will be a useful strategy in preventing carcinogenesis of the pre-cancerous hyperplastic endometrium. Discovering novel treatments that prevent the progression of EH to EC may prove useful in high-risk women, such as obese patients diagnosed with endometrial hyperplasia.

Within our cohort, patients with EH had significantly higher BMI compared with pre-menopausal (*p* = 0.0061), post-menopausal (*p* < 0.0001), and EC (*p* = 0.0008) patient groups. Other studies have found that obesity increases the risk of mortality in EC by two- to six-fold [22]. Therefore, by establishing nucleolin’s role in EH and EC and comparing effects between women of different BMI on disease progression, mortality, and recurrence, *NCL* may be studied as a potential target for both cancer prevention and anti-cancer therapies in this particular at-risk EH patient group. Such treatment may have major clinical relevance for women diagnosed with EH within the reproductive period, potentially allowing targeted fertility-sparing management in those who wish to retain their uterus, and thus, their fertility.

In their study of TCGA data, Lin et al. found higher *NCL* mRNA expression to significantly correlate with serous endometrial carcinoma (*p* < 0.001), advanced stage (*p* = 0.029), and grade 3 endometrioid EC (*p* < 0.001), all of which had a poor prognosis [14]. Statistical analysis in our study revealed an association of *NCL* mRNA levels with EC, where healthy PM endometrium expressed significantly higher levels of *NCL* mRNA compared with EC (*p* < 0.01). Interestingly, this finding does not correlate with our immunohistochemical analysis of the nucleolin protein levels, where no significant difference in nucleolin quick score was observed between PM and EC samples. This may be explained by the different entities measured by the two techniques, for example, mRNA vs. protein, and immunohistochemistry staining analysis did not directly quantify nucleolin protein levels, but semi-quantitatively assessed the proportion and staining intensity of nucleoli. Furthermore, mRNA levels do not have a linear relationship with protein levels. This mapping can be affected by parameters and conditions specific to *NCL*, thereby necessitating further research into factors influencing its translation in the future. Fortelny et al. further support this notion by observing mRNA levels to be a poor predictor of the abundance of the protein, as proteins do not have a one-to-one ratio with their corresponding mRNA [23]. Mehra et al. indeed state that mRNA changes do not equate with changes in corresponding proteins, which ultimately govern cellular function [24], or in the case of our study of EC, cellular dysfunction. Instead, Mehra et al. invite the integration of polysome size, translation elongation, and protein stability in the future study of mapping between mRNA and protein levels [24].

Existing evidence on the prognostic value of *NCL* points to higher mRNA expression associated with a worse OS (*p* = 0.0001) and DFS (*p* = 0.006) [14]. Interestingly, conversely, *NCL* protein expression in endometrioid EC shows the opposite trend, with higher *NCL* protein expression levels in the nucleus carrying a better DFS than those with low nuclear *NCL* immunoexpression (*p* = 0.001) [14]. In our study, although not statistically significant, there was an obvious trend displaying worse OS in women with ECs expressing high nucleolar *NCL* protein compared with women with low nucleolar *NCL* protein expression (*p* = 0.05). In patients with HGEC tumours, higher nucleolar *NCL* protein immunoexpression carried a worse OS than those demonstrating lower nucleolar *NCL* expression, with a median survival of 15 months in *NCL* quick score >6 (*p* = 0.01). The data did not show a statistically significant difference for DFS (*p* > 0.05). Our study therefore supports the overall survival analysis of TCGA mRNA data in the study by Lin et al., and further indicates that *NCL* may be an important marker in the stratification of high-risk groups in HGEC, enabling the careful selection of those patients for potential *NCL* targeted therapy to improve OS. However, larger studies are needed to confirm our findings, which may allow the input of *NCL* expression levels in predictive models and allow patients with HGEC expressing low *NCL* to be spared unnecessary adjuvant treatment, thereby improve patient quality of life.

Metastasis remains to be one of the most important prognostic factors in EC. Large population-based case studies have proven that EC OS and cancer-specific survival (CSS) are largely influenced by metastasis, with both 3-year OS and CSS at a striking 0% in multi-site metastatic disease [25]. EC metastasis can be classified into three modes: lymphatic, intra-abdominal, and distant organ metastasis to the lung, liver, bone, and brain, with the most common metastatic disease being lymph node metastasis [25]. For the first time, our study has examined the difference in nucleolar *NCL* levels in primary endometrial tumours and their secondary metastatic lesions. An overall higher nucleolar expression of *NCL* in metastatic lesions was observed when compared to primary tissue in the same patient (*p* = 0.04). These findings suggest *NCL* expression may be a useful molecular marker in molecular targeted therapies, allowing for the prevention of metastatic disease and improving patient OS and CSS.

In this study, we aimed to assess *NCL* mRNA levels and immunolocalisation in a local cohort consisting of healthy, hyperplastic, EC, and metastatic samples. We confirm our hypothesis of differential *NCL* expression in EC, thus informing future research of a potential novel therapeutic target in EH, HGEC, and metastatic disease. Currently, there are no approved molecular targeted therapies for endometrial cancer [26]. Rapidly advancing knowledge in the field of translational medicine and molecular biology has suggested that *NCL* may be targeted via miRNA, aptamers, and peptides, proving promising for future anti-cancer therapy [6]. A key priority should therefore be to verify the effectiveness of these therapies in EC, via clinical trials. This may allow for the use of *NCL* in the molecular classification of EC, guiding decision-making in routine clinical practice.

As an extension to the ex vivo study, we also explore the role of *NCL* and NAGs in EC by examining the RNA sequencing data of the TCGA uterine cancer cohort through in silico analysis. Overall, 197 NAGs were found to be differentially expressed in the TCGA EC samples, except for the genes coding T cell receptor (*TCR*). *TCR* is a protein complex that controls the development, differentiation, and survival of T cells [27]. Its structure is highly intricate, coded by four genes, *TCRα*, *TCRβ*, *TCRδ*, and *TCRγ* [28]. The TCGA database did not contain RNA levels for *TCRα*, *TCRβ*, *TCRδ*, and *TCRγ*, and so their expression in EC could not be analysed. This suggests that other microarray datasets should be analysed in the future to allow for an understanding of TCR’s contribution to endometrial carcinogenesis.

Our bioinformatics analysis further revealed *NCL* to be the most highly upregulated gene in the carcinosarcoma subtype of EC (log_2_ fold change = 6022.939, adjusted *p* value = 0.00609). Carcinosarcoma (CS) is an aggressive subtype of endometrial tumour, presenting with metastatic disease in 60% at diagnosis [29]. Despite surgical treatment and adjuvant therapy, it is thought to recur in over 50% of patients, therefore necessitating an improvement in management strategies. Urgent development of novel targeted treatment to prevent disease recurrence and formation of metastatic lesions is needed. For that reason, *NCL* may prove to be a useful marker of the CS subtype of EC, allowing targeted aggressive adjuvant therapy.

When comparing the expression of NAGs between EC and healthy adjacent endometrium, multiple genes were differentially expressed. The top five upregulated genes identified were *S100A11*, *LMNB2*, *SCRIB*, *CCNB1,* and *SFRS2*. All five genes were present in both, samples exposed and unexposed to any hormonal, neoadjuvant, or radiation therapy, thereby signifying their importance. Furthermore, when validating our results with an external GEO dataset of EC, we found that *CCNB1*, *S100A11*, and *LMNB2* were upregulated in the GSE17025 dataset. This result denotes the significance of their role in endometrial carcinogenesis, allowing future laboratory studies to investigate their expression levels in EC. Available wet-lab data on *CCNB1* validate our results by existing studies noting *CCNB1* upregulation in EC, with suggested involvement of Cyclin B1 in the proliferation and reduced differentiation of EC [30].

On the other hand, our study has highlighted several novel potential biomarkers associated with *NCL* which may have a role in EC, including *S100A11* and *LMNB2*. *S100A11* is a member of S100 proteins, with a role in the regulation of cell growth, differentiation, and apoptosis [31]. Its involvement in carcinogenesis is well established, with high expression correlated to tumour formation, migration, and metastasis in renal cell, hepatocellular, prostate, breast, cervical, and ovarian carcinomas [32,33,34,35,36,37]. Liu et al. have isolated its presence in human endometrium in relation to reproductive failure [38]; however, there are no current studies exploring *S100A11*’s role in EC. Interestingly, Liu et al. found *S100A11* knockdown to reduce embryo implantation rate, implying its downregulation may be implicated in reproductive failure. Our study found *S100A11* to be upregulated in EC when compared to adjacent healthy tissue, with unique upregulation in endometrioid tumours, and specific high expression in G1 and G3 endometrioid tumours. Furthermore, we found *S100A11* upregulation to also be unique to LGEC in both TCGA and GEO datasets. Therefore, together with the findings of Liu et al., our study suggests a role of *S100A11* in the human endometrium, indicating the future examination of *S100A11*’s functional role in endometrial pathologies such as endometrioid EC.

Furthermore, our study is the first to find significant upregulation of *LMNB2* in EC. *LMNB2* is a lamin protein known to regulate nuclear stability and gene expression [39], with noted upregulation in breast, colorectal and oesophageal cancers [40,41,42]. Our in silico study notes *LMNB2* to be highly expressed in all EC subtypes, with unique upregulation in HGEC and G3 endometrioid cancer. This suggests *LMNB2* may be involved in nuclear instability and the progression of EC into HGEC. Therefore, future studies should aim to explore *LMNB2* as a potential prognostic marker in HGEC, allowing for potential risk stratification in high-grade diseases.

The most significantly downregulated genes in EC were *SVIL*, *RERG*, *ITGAV*, *RASL12*, *GEM*, and *CDH5*. All five genes were present in both samples exposed and unexposed to any hormonal, neoadjuvant, or radiation therapy; however, these genes were not identified as downregulated in our external GSE17025 dataset. This means that our data on downregulated genes may need to be interpreted with caution, and further studies should aim to compare their expression with other large cohorts to determine their true role in EC development. Nevertheless, despite not being differentially expressed on external validation, research into these genes may bring new insights into the pathogenesis of EC, as recent work shows their involvement in cancer development. For example, *SVIL* is intricately involved in tumour angiogenesis in liver cancer [43], whilst *ITGAV* silencing has proven to inhibit the cell proliferation and invasion of breast cancer cell lines [44] and *CDH5* has been demonstrated as a biomarker of metastatic breast cancer [45]. The prognostic value of all DEGs persisting despite exposure to hormonal, neoadjuvant, or radiation therapy determined that *RERG*, the second most highly downregulated DEG in our analysis, was an unfavourable molecular prognostic marker in EC. Similarly, *E2F1* and *CCNA2* were upregulated in the GSE17025 and TCGA datasets, demonstrating to have prognostic relevance in The Human Protein Atlas. Therefore, we invite future research into these identified gene alterations, with particular focus on verifying the role of *S100A11*, *LMNB2*, *RERG*, *E2F1* and *CCNA2* in EC, thereby bringing new insights into the pathogenesis of the disease.

The joint evidence-based guidelines of the European Society of Gynaecological Oncology (ESGO), European Society for Radiotherapy and Oncology (ESTRO), and European Society of Pathology (ESP) for the management of EC recommended using a panel of molecular markers to classify EC in addition to the well-known, clinicopathologic features such as myometrial invasion, histotype or lymph vascular space invasion [46]. The markers already in use are not considered to be fully comprehensive and have room for improvement. Given the extent of the interaction nucleolin and NAGs have with the other known prognostic molecular markers, (e.g., POLE, TP53, MSH6, PMS2, KRAS, PTEN, and L1CAM, shown in Supplementary Figure S4), it would be interesting to see whether the NAGs with a prognostic value can be integrated with other existing clinical and molecular classifiers when deliberating the need for adjuvant chemotherapy (high-grade/high-risk disease).

A strength of our qPCR study is that our qPCR cohort consisted of all EC subtypes thereby making our results representative and generalisable to all EC. Our study also used 4 observers in the quantification of nucleolar *NCL* immuno-expression, demonstrating a high agreement, and increasing the reliability of our data. The use of an external EC cohort from the GEO database also further validate our results after differentially expressed NAGs were scrutinised in both exposed and unexposed cohorts. One limitation of our study was that small sample sizes were used in qPCR and IHC. This may explain the differences in our findings to that of Lee et al.; however, future studies should aim to further investigate *NCL*’s expression in EC with larger sample sizes to determine *NCL*’s true role in EC carcinogenesis. The use of only immunohistochemistry to quantify *NCL* protein expression levels in EC may be another limitation. Further research seeking to confirm our data should rectify these limitations. Although not all identified DEGs were differentially expressed in the external GEO dataset, this may be explained by the use of different control samples, where TCGA data utilised an EC healthy adjacent normal control tissue, whilst GSE17025 employed healthy PM samples. Additional DEGs identified as upregulated or downregulated in TCGA data may have been influenced by concurrent hyperplasia in adjacent samples, and so may not be a true display of differential gene expression in EC. This may suggest that TCGA datasets alone should not always be used as gold-standard in bioinformatic analysis for EC research, but future in silico studies should employ TCGA data in parallel with several other representative publically available EC datasets to draw clinically relevant and more accurate conclusions.

## 4. Materials and Methods

### 4.1. Endometrial Tissue Samples

Endometrial samples were obtained from one hundred and fifty-seven women undergoing gynaecological surgery between 2009 and 2017 at Liverpool Women’s Hospital and Lancashire Teaching Hospitals Trusts. Samples were collected from women who were not exposed to any hormonal, neoadjuvant, or pelvic radiation therapy prior to surgery. Control group consisted of 38 women undergoing hysterectomy or laparoscopy for benign gynecological conditions (e.g., laparoscopic sterilisation, prolapse surgery, menstrual disorders, vaginal bleeding who did not have endometriosis, cancer or a known endometrial pathology). Pre-menopausal women included were in the proliferative phase of the cycle. EC and hyperplasia groups consisted of 98 and 21 women, respectively, undergoing hysterectomy with a diagnosis of endometrial hyperplasia or endometrial cancer, and their surgery was completed using laparoscopic route or laparotomy. The endometrial samples were collected using a Pipelle endometrial sampler. Basic demographic information was obtained prospectively on-site at the time of sample collection. Histological subtype and cancer grade were assigned according to FIGO guidelines [47]. This study’s cohort consisted of 21 samples with endometrial hyperplasia, 98 with endometrial cancer, 35 metastatic lesions and 38 samples of healthy endometrium to be used as control tissue, with further details presented in Table 1. 21 of the EC patients had concurrent endometrial hyperplasia at the time of surgery, whilst 27 had metastatic disease. Ethical approval was granted by the Welsh and Scottish Ethics Committees (19/WA/0271 and 19/SC/0449) and Cambridge Adult Research Ethics Committee (CREC 10/H0308/75).

### 4.2. Real-Time qPCR

RNA was extracted from tissue samples and EC cell lines with TRIzol^®^ Plus RNA Purification Kit (Thermo Scientific, Loughborough, UK). Purified RNA was DNAse treated with 1 unit of DNAse (Promega, Southampton, Hampshire, UK) per 1–5 μg of RNA. Total RNA was quantified by FLUOstar Omega microplate reader (BMG LABTECH, Aylesbury, Bucks UK) and 1 μg was reverse transcribed with iScript™ cDNA Synthesis Kit (Bio-Rad Laboratories Ltd., Hemel Hempstead, Hertfordshire, UK) as per manufacturer protocol. 1 μL of cDNA was amplified in triplicate for 40 cycles in a final reaction volume of 10 μL using iTaq Universal SYBR Green Supermix (Bio-Rad Laboratories Ltd., Hemel Hempstead, Hertfordshire, UK) and Biorad CFX Connect Real-Time System (Bio-Rad Laboratories Ltd., Hemel Hempstead, Hertfordshire, UK). Ishikawa EC cell line (ISK) was run as an internal control. The primer sequences are seen in Table A1, Appendix A. No template and no reverse transcriptase controls were included for each target in each experiment. The ΔΔCT method was used to calculate transcript expression of nucleolin relative to reference genes *IPO8*, *PPIA*, and *MRPL19* and normalised to ISK using Biorad CFX manager (version 3.1, Bio-Rad Laboratories Ltd., Hemel Hempstead, Bucks, UK.

### 4.3. Immunohistochemistry

Standard immunohistochemical techniques were used to perform nucleolin staining of the endometrial tissue samples. In brief, 3 μm thick sections of endometrial specimens were baked at 60 °C for 1 h using Section Dryer Model E28.5 (Thermo Scientific, Loughborough, UK), followed by deparaffinisation and rehydration. To restore epitopes, samples were immersed in a pressure cooker containing 10 mM citrate buffer at pH 6 for 2 min. Endogenous peroxidase was quenched in 0.3% H_2_O_2_/Tris-buffered saline (TBS) (Sigma-Aldrich, Poole, Dorset, UK). After washing with TBS, 2.5% Normal Horse Serum Blocking Solution (Vector Laboratories, 2B Scientific Ltd., Upper Heyford, Oxfordshire, UK) was used to block non-specific protein binding. Sections were then incubated with anti-nucleolin rabbit polyclonal antibody (ab22758, Abcam, Cambridge, Cambs., UK) at 1:32,000 dilution overnight at 4 °C in a humidifying chamber. A matching control of anti-rabbit IgG polymer at 1:10,000 replaced the primary antibody as a negative control. Detection of the antibody was with ImmPRESS horseradish-peroxidase-conjugated anti-rabbit IgG polymer (Vector Laboratories, 2B Scientific Ltd., Upper Heyford, Oxfordshire, UK), whilst visualisation was with ImmPACT DAB chromagen (Vector Laboratories, 2B Scientific Ltd., Upper Heyford, Oxfordshire, UK), which yielded brown staining. The sections were counterstained using filtered Shandon Gill 2 Haematoxylin (Thermo Scientific, Loughborough, Leics., UK), dehydrated, and cleared in xylene, prior to mounting with Shandon Consul-Mount (Thermo Scientific, Loughborough, Leics., UK).

All slides were visualised on Aperio ImageScope (version 12.4.3, Leica Biosystems Imaging, Deer Park, IL, USA) at ×40 magnification, following scanning on Leica Aperio CS2 Slide Scanner (Leica Biosystems Imaging, Milton Keynes, Bucks, UK). Sections were scored blind by four independent observers prior to breaking the code. The scoring system included calculating the proportion of nucleoli stained in different intensities. This was performed semi-quantitatively via the modified quick score method [48]. Each section was evaluated for proportion stained (0: 0% stained; 1: 1–25% stained; 2: 26–50% stained; 3: 51–75% stained; 4: 76–100% stained) and intensity (0: no staining; 1: weak staining; 2: moderate staining; 3: strong staining), yielding a score where the intensity and proportion scores were multiplied, then summed to give a final quick score in a range of 0–12. For example, if in a section it was found that 25% was weakly stained (1 × 1 = 1), 50% moderately stained (2 × 2 = 4), and 25% strongly stained (1 × 3 = 3), this would give a final quick score of 1 + 4 + 3 = 8. Findings were tabulated and any disagreements over 2 points in scoring were discussed before calculating a mean quick score for each section. Code was then broken.

### 4.4. In Silico Study

#### 4.4.1. Identification of Nucleolin Associated Genes (NAGs)

NAGs were identified via Search Tool for the Retrieval of Interacting Genes/Proteins (STRING) and QIAGEN Ingenuity Pathway Analysis (IPA) [49,50,51,52]. STRING detected functional and physical protein associations in humans by exploring all active interaction sources, except for text mining. The interaction score was set at the highest confidence (0.900) with a maximum of 500 1st shell interactors. NAGs identified via IPA were included via direct interactions found upstream or downstream in humans in all data sources. Genecards [53,54] were utilised to identify aliases for NAGs. Duplicates were manually removed.

#### 4.4.2. TCGA Cohort

RNA sequencing and demographic data were previously obtained from The Cancer Genome Atlas (TCGA) database with Broad Genome Data Analysis Centre [55]. Uterine Corpus Endometrial Carcinoma (TCGA-UCEC) and Uterine Carcinosarcoma (TCGA-UCS) datasets were utilised in this study. Samples exposed to any hormonal, neoadjuvant, or radiation therapy were excluded from the main sample set of this study and only used to identify NCL and Differentially Expressed Genes (DEGs) which are common to both exposed and unexposed samples signifying their importance.

#### 4.4.3. Identification of Differentially Expressed Genes in TCGA Dataset

Normalised TCGA RNASeqV2 data of NAGs were analysed via iDEP.91 [56,57]. Cut-off criteria included a False Discovery Rate (FDR) <0.01 and Fold Change (FC) >2. Comparisons in DEGs were performed between cancer and healthy adjacent endometrium, across histotypes of EC, and EC grades. Volcano plots and heatmaps were created in iDEP.91, and Venn diagrams in the Bioinformatics and Evolutionary Genomics tool [58]. Prognostic value in EC of genes common to both exposed and unexposed samples was determined via exploring data in The Human Protein Atlas [59,60]. Diagrams visualising Protein–protein interaction (PPI) networks were constructed in Cytoscape version 3.8.2 [61] with available data from STRING and IPA.

#### 4.4.4. Biological Processes and Pathway Analyses

Enrichr [62,63,64,65] was utilised to perform analysis of Gene Ontology (GO) biological processes and Kyoto Encyclopedia of Genes and Genomes (KEGG) pathways of DEGs found to be prognostic in EC.

#### 4.4.5. Validation of Differentially Expressed NAGs

Gene Expression Omnibus (GEO) [66] was explored to find datasets similar to the TCGA cohort. The selected dataset was analysed with GEO2R [18] for NAGs with cut off criteria of Log2FC > 1 and an adjusted *p* value < 0.05.

#### 4.4.6. STRING Network of Known Molecular Markers for EC and NAGs

Known molecular markers of endometrial cancer were identified using a PubMed search and included in a STRING network with the 11 genes with prognostic value persisting despite hormonal, radiation, or neoadjuvant therapy.

### 4.5. Statistical Analysis

The statistical programme GraphPad Prism (version 5.0, GraphPad Software, San Diego, CA, USA) was used for all wet lab data calculations. Quick scores, normalised NCL gene expression, and demographic details were analysed using non-parametric tests (Kruskall–Wallis/Dunn’s post hoc or Mann–Whitney U-test). For comparison between paired values, the Wilcoxon Matched Pairs test was used. Overall Survival (OS) and Disease-Free Survival (DFS) were analysed by Kaplan-Meier survival curves with a cut-off score of 6 for NCL quick score, and compared statistically via a Log-rank Mantel-Cox Test. A value of *p* < 0.05 was considered significant.

## 5. Conclusions

In conclusion, we have demonstrated, for the first time, the involvement of nucleolin in endometrial hyperplasia, HGEC, and metastatic disease. Our in silico analysis has uniquely revealed a possible role of *S100A11*, *LMNB2*, *RERG*, *E2F1*, and *CCNA2* in endometrial carcinogenesis, allowing future research to focus on their involvement in the pathogenesis of the disease, with an ultimate focus on risk stratification in high-grade EC.

## Figures and Tables

**Table 1:** Patient demographic features. **Age**: Pre-menopausal vs. post-menopausal *p* < 0.0001. Pre-menopausal vs. endometrial hyperplasia *p* < 0.0001. Pre-menopausal vs. Endometrial Cancer *p* < 0.0001. Endometrial hyperplasia vs. endometrial cancer *p* = 0.0007. **BMI**: Endometrial hyperplasia vs. pre-menopausal *p* = 0.0061. Endometrial hyperplasia vs. post-menopausal *p* < 0.0001. Endometrial hyperplasia vs. endometrial cancer *p* = 0.0008.

Study Group	Number of Patients	Age (Years) Median (Range)	BMI (kg/m^2^) Median (Range)
**Healthy Control**	38	56.5 (30–85)	26.8 (20–52.2)
Pre-menopausal	15	44 (30–47)	27.8 (21.6–52.2)
Post-menopausal	23	62 (52–85)	25.2 (20–39.6)
**Endometrial Hyperplasia**	21	57 (37–74)	37.8 (23.6–63.1)
**Endometrial Cancer**	98	67 (33–89)	30 (20.2–51.4)
**Low Grade EC**	39	60 (33–88)	30.6 (21.6–48.4)
Grade 1 Endometrioid Carcinoma	22	58 (33–88)	31.2 (21.6–47.6)
Grade 2 Endometrioid Carcinoma	17	64 (41–79)	28.9 (41–48.4)
**High Grade EC**	59	73 (52–89)	29.6 (20.2–51.4)
Grade 3 Endometrioid Carcinoma	14	70 (54–83)	28.6 (23.9–42.7)
Serous Carcinoma	13	78 (60–87)	29.6 (23.4–38.7)
Carcinosarcoma	20	78 (58–89)	26 (20.2–51.4)
Clear Cell Carcinoma	11	64 (52–82)	30.3 (26.6–39)
Mixed Endometrioid and Clear Cell	1	83	26.8

## Data Availability

Data are contained within the article or Supplementary Material.

## Supplementary Materials

The following supporting information can be downloaded at: https://www.mdpi.com/article/10.3390/ijms23116228/s1.

## Appendix A

**Table A1:** Primer sequences used in qPCR.

Gene Name	Sequences (5′ ≥ 3′)	Product Size (bp)	Efficiency (%)
IPO8	Fwd: AGGATCAGAGGACAGCACTGCA	102	97
	Rev: AGGTGAAGCCTCCCTGTTGTTC		
PPIA	Fwd: AGACAAGGTCCCAAAGAC	118	100
	Rev: ACCACCCTGACACATAAA		
MRPL19	Fwd: CAGGAAGAGGACTTGGAGCTAC	137	101.5
	Rev: GCTATCATCCAGCCGTTTCTCTA		
NCL	https://www.bio-rad.com/en-uk/prime-pcr-assays/assay/qhsaced0043572-primepcr-sybr-green-assay-ncl-human	160	94
