# Supplementary material for: Role of Nucleolin in Endometrial Precancerous Hyperplasia and Carcinogenesis: Ex Vivo and In Silico Study
Source: Int J Mol Sci. 2022 Jun 2;23(11):6228. doi: 10.3390/ijms23116228 (PMC9181237; doi:10.3390/ijms23116228)
Supplement: Supplementary file 1 [file ijms-23-06228-s001.zip › Supplementary figures.pdf]

International Journal of
Molecular Sciences

MDPI

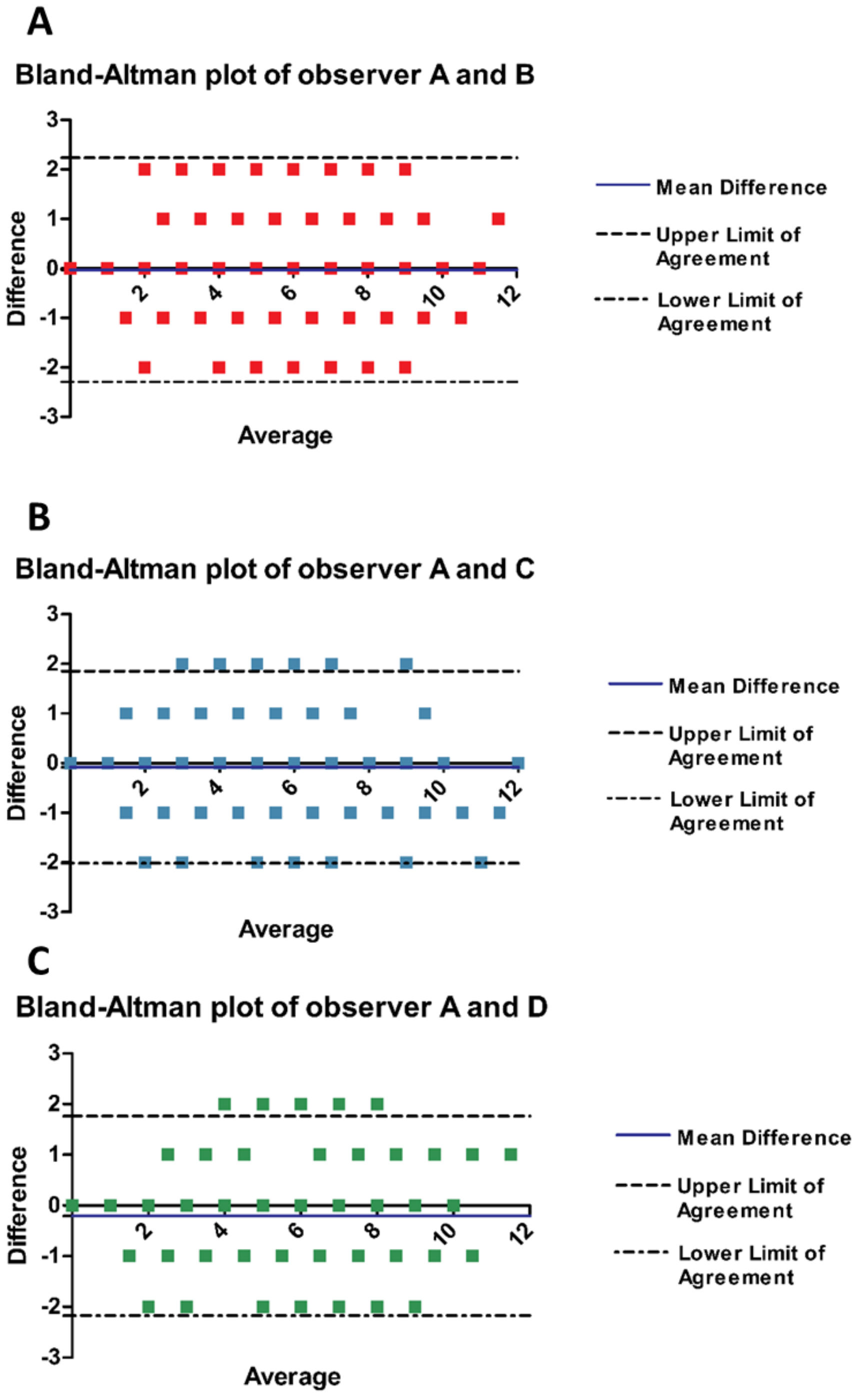

Figure S1. Bland-Altman graphs evaluating the agreement between mean nucleolar nucleolin quickscores amongst the four observers. (A) Bland-Altman plot of observer A and B: Mean Difference = -0.027; Upper Limit of Agreement = 2.239; Lower Limit of Agreement =-2.294. (B) Bland-Altman plot of observer A and C: Mean Difference = -0.082; Upper Limit of Agreement = 1.851; Lower Limit of Agreement = -2.015. (C) Bland-Altman plot of observer A and D: Mean Difference = -0.206; Upper Limit of Agreement = 1.760; Lower Limit of Agreement = -2.171.

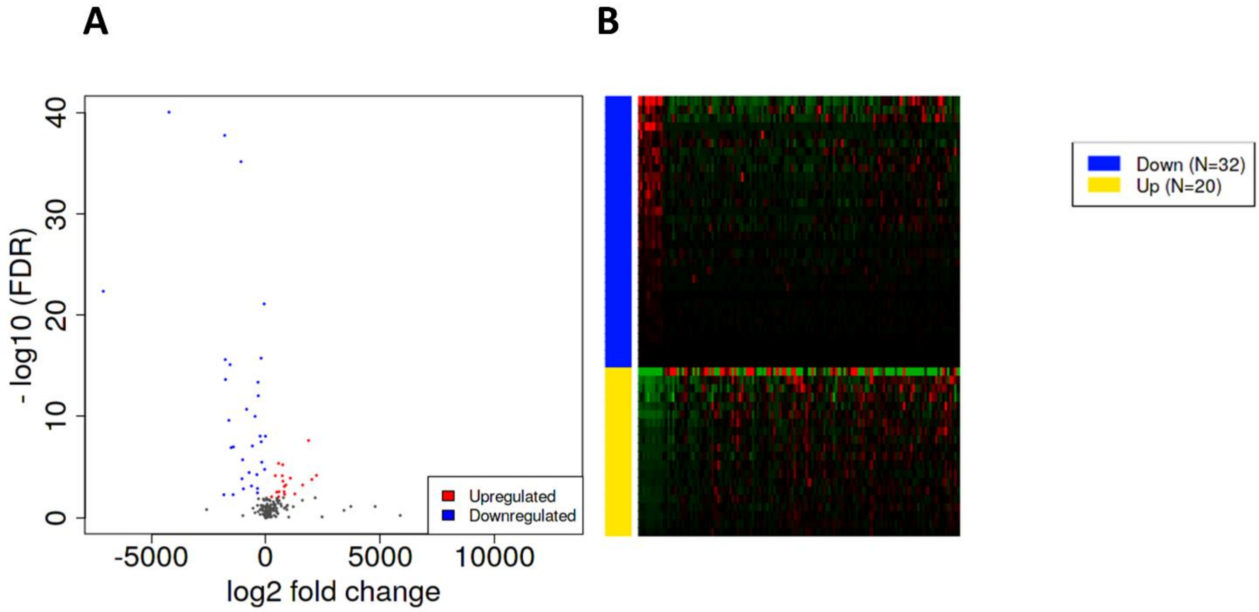

Figure S2. Volcano plot (A) and heatmap (B) of Differentially Expressed Genes in Endometrial Cancer compared to Healthy Adjacent Tissue.

A

Grade	Upregulated DEGs	Downregulated DEGs
**G1 / G2 / G3**	*N/A*	*RERGL, RERG, PIWIL2, EIF2C4, ITGA5, REM1, RASL12, DIRAS1, ZNF532, ITGB3, GEM, NKIRAS1, SFMBT2, MRAS, CDH5, RASD2, C1QTNF4, USP51, RAP2C, RAP1B, SELP, RRAS, RAP1A, RASL11A, SVIL, THRA*
**G1 / G3**	*S100A11*	*DIO3*
**G2 / G3**	*CHAF1A, CCNB1, HIST1H3H*	*MAX, RNASEL, BCL2, VTN, RALB*
**G2**	*MDM2, REM2, HIST1H3D*	*N/A*
**G3**	*NKIRAS2, C6ORF153, NOP58, FUS, DDX49, DKC1, IMP4, RRP9, EIF1AD, NRAS, AURKB, E2F1, RPA3, DHX37, CCNA2, NOP14, DCAF13, SCRIB, SFRS2, UTP6, BMS1, LMNB2, IMP3*	*UTP14C, SP1, TACR1, NAP1L1, SIN3B*

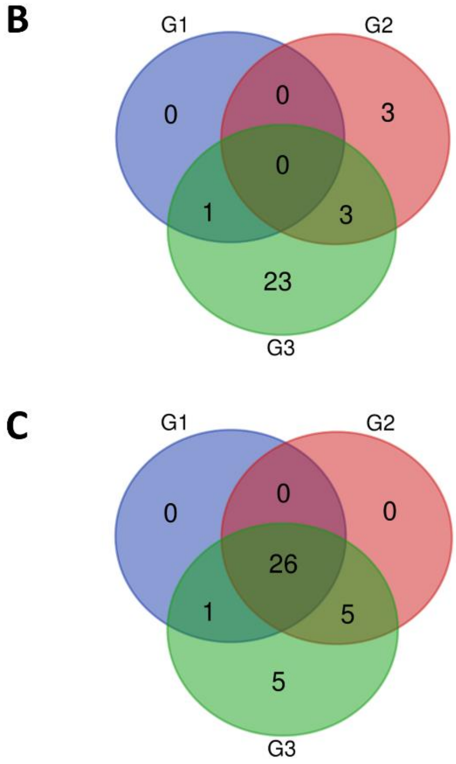

Figure S3. *In Silico* analysis of G1, G2 and G3 endometrioid cancer tumours. (A) Table of upregulated and downregulated DEGs in G1, G2 and G3 endometrioid tumours. Venn diagrams of (B) upregulated and (C) downregulated DEGs in G1, G2 and G3 endometrioid tumours.

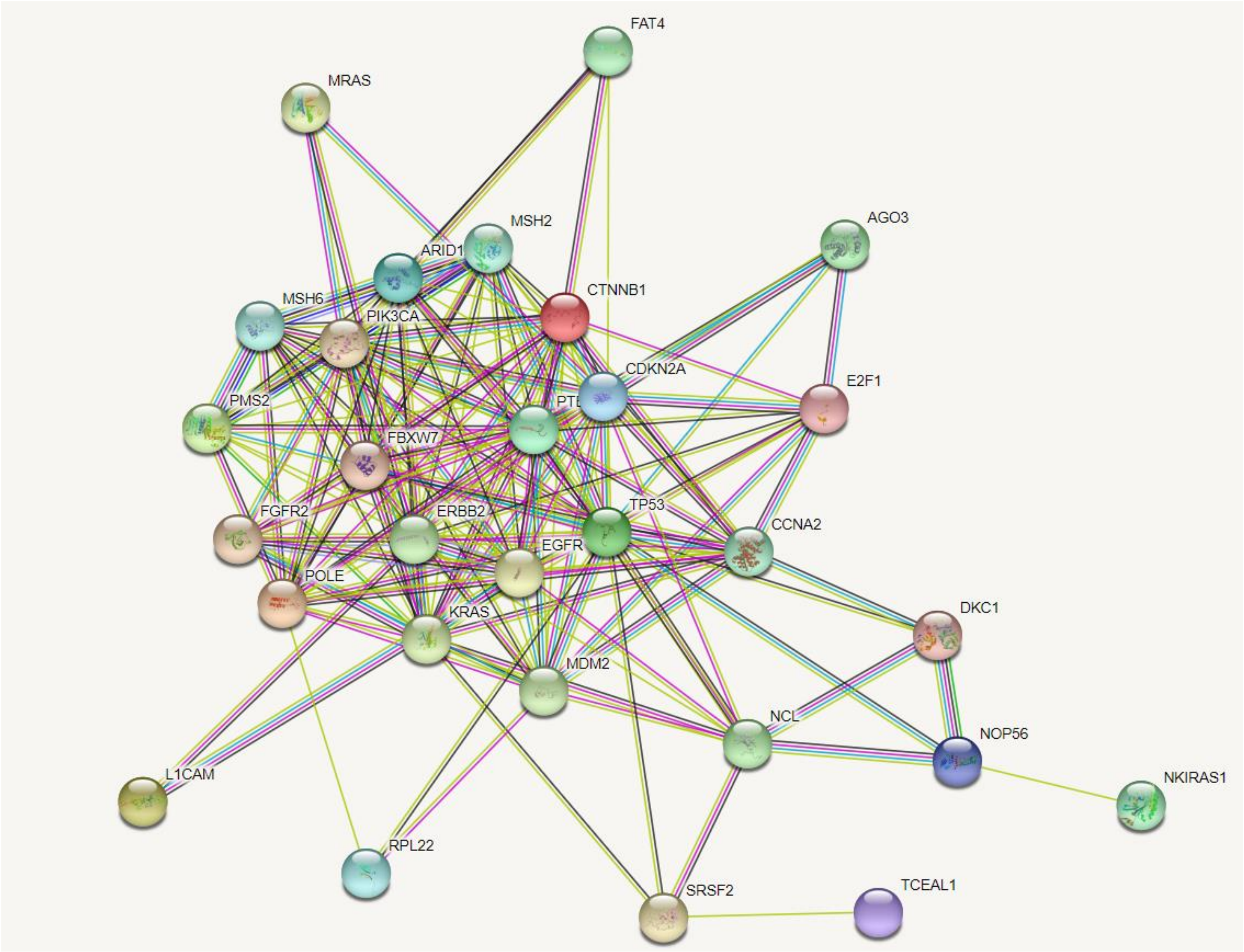

Figure S4. STRING network showing the interaction between nucleolin and the NAGs prognostic for EC irrespective of exposure to hormonal, radiation or neoadjuvant therapy (CCNB1, SFRS2, E2F1, CCNA2, DKC1, NOP56, MRAS, NKIRAS1, EIF2C3, MDM2) and known molecular markers of EC (POLE, PTEN, L1CAM, PIK3CA, KRAS, TP53, TCEAL1, CDKN2A, CTNNB1, ARID1A, FBXW7, ERBB2, EGFR, FGFR2, PMS2, MSH2, MSH6, RPL22, FAT4, DMD). Only 4 of the 13 identified NAGs (RERG, ZNF532, EIF1AD and TRIM3) do not form a part of this network.
